# A Hybrid Sales Forecasting Scheme by Combining Independent Component Analysis with K-Means Clustering and Support Vector Regression

**DOI:** 10.1155/2014/624017

**Published:** 2014-06-17

**Authors:** Chi-Jie Lu, Chi-Chang Chang

**Affiliations:** ^1^Department of Industrial Management, Chien Hsin University of Science and Technology, Taoyuan County 32097, Taiwan; ^2^School of Medical Informatics, Chung Shan Medical University, Information Technology Office, Chung Shan Medical University Hospital, Taichung City 40201, Taiwan

## Abstract

Sales forecasting plays an important role in operating a business since it can be used to determine the required inventory level to meet consumer demand and avoid the problem of under/overstocking. Improving the accuracy of sales forecasting has become an important issue of operating a business. This study proposes a hybrid sales forecasting scheme by combining independent component analysis (ICA) with K-means clustering and support vector regression (SVR). The proposed scheme first uses the ICA to extract hidden information from the observed sales data. The extracted features are then applied to K-means algorithm for clustering the sales data into several disjoined clusters. Finally, the SVR forecasting models are applied to each group to generate final forecasting results. Experimental results from information technology (IT) product agent sales data reveal that the proposed sales forecasting scheme outperforms the three comparison models and hence provides an efficient alternative for sales forecasting.

## 1. Introduction

Sales forecasting is one of the most important tasks in many companies since it can be used to determine the required inventory level to meet consumer demand and avoid the problem of under/overstocking. In addition, sales forecasting can have implications for corporate financial planning, marketing, client management, and other areas of business. Therefore, improving the accuracy of sales forecasting has become an important issue of operating a business.

Recently, many researches have been conducted on the study of sales forecasting in various industries such as clothing [1, 2], fashion [3–6], book [7], electronics [8, 9], and automotive industry [10]. However, there is little research centered on sales forecasting for the information technology (IT) industry. Lu and Wang [11] developed a hybrid sales forecasting model for computer dealer. Lu [12] proposed a two-stage sales forecasting model by integrating multivariate adaptive regression splines and support vector regression (SVR) for computer products. Lu et al. [13] employed eleven forecasting methods for predicting sales of computer wholesalers. With technological advancements and rapid changes in consumer demand, computer products are characterized by product variety, rapid specification changes, and rapid price declines. These factors have made sales forecasting in the IT industry, especially in IT product agents, an important but difficult task. This paper focuses on sales forecasting for IT product agents in light of the important role they play in the IT industry by distributing IT products to retailers and industry customers.

In order to improve the forecasting performance, many studies have proposed different kinds of forecasting models based clustering algorithms [14–16]. For a given input data, the forecasting model first uses a clustering algorithm to partition the whole input space into several disjoint regions. Then, for each partition, the forecasting method is constructed to produce the output. Since the partitioned regions have more uniform/stationary data structure than that of the whole input space, it will become easier for constructing effective forecasting model.

However, the existing clustering-based forecasting models usually directly use the observed original values of prediction variables for data clustering [14–16]. But some underlying factors may not be directly observed from the original data. Therefore, if the underlying/interesting information that cannot be observed directly from the observed original data can be revealed through transforming the input space into the feature space with a suitable feature extraction method, the performance of the clustering-based forecasting model can be improved by using the features as inputs to produce more effective clustering results. Therefore, independent component analysis (ICA), a novel statistical signal processing technique, is used in this research.

ICA model was proposed to find the latent source signals from observed mixture signal, without knowing any prior knowledge of the mixing mechanisms [17]. It is aimed at extracting the hidden information from the observed data where no relevant data mixture mechanisms are available. ICA has been reported to have the capability of extracting the distinguishability information from the time series data [11, 17, 18]. Back and Weigend [19] used ICA to exact the features of the daily returns of the 28 largest Japanese stocks. The results showed that the dominant ICs can reveal more underlying structure and information of the stock prices than principal component analysis. Kiviluoto and Oja [20] employed ICA to find the fundamental factors affecting the cash flow of 40 stores in the same retail chain. They found that the cash flow of the retail stores was mainly affected by holidays, seasons, and competitors' strategies.

In this study, a hybrid sales forecasting scheme by combining ICA with K-means clustering and support vector regression (SVR) is proposed. In the proposed scheme, first, the ICA model is applied to the input data to estimate features. Then, the features are used as inputs of K-means clustering algorithm to group the input data into several disjoined clusters. As K-means is one of the most used clustering algorithms [21], it is utilized in this study. In the final step of the proposed scheme, for each cluster, the SVR forecasting model is constructed and the final forecasting results can be obtained. This study considers the SVR as the predictor due to its great potential and superior performance in practical applications [18, 22]. SVR based on statistical learning theory is a novel neural network algorithm and has been receiving increasing attention for solving nonlinear regression estimation problems [14, 15, 18]. This is largely due to the structure risk minimization principles in SVR, which has greater generalization ability and is superior to the empirical risk minimization principle as adopted by traditional neural networks. SVR has been successfully and widely used in various forecasting problems such as electric load forecasting [23–25], wind speed [26], traffic flow [27, 28], and financial time series forecasting [29–35].

There are only very few articles utilizing ICA and SVR in constructing clustering-based sales forecasting model. Lu and Wang [11] combined ICA, growing hierarchical self-organizing maps (GHSOM), and SVR to develop a clustering-based sales forecasting model. In their approach, the principal component analysis (PCA) was used as the preprocessing method of ICA for dimension reduction. However, using PCA to reduce the dimension of the original data before ICA may lose some useful information for feature extraction. Moreover, the GHSOM is a complex and time consuming model. Since the proposed method does not use PCA for dimension reduction and utilizes the simple, fast, and well-known K-means method for data clustering, it is believed that the proposed clustering-based sales forecasting approach differs from the method proposed by Lu and Wang [11] and hence provides an ideal alternative in conducting sales forecasting for computer wholesalers.

The rest of this paper is organized as follows.  Section 2 gives a brief introduction about independent component analysis and support vector regression. The proposed clustering-based sales forecasting model is thoroughly described in Section 3.  Section 4 presents the experimental results from a computer wholesaler sales data. The paper is concluded in Section 5.

## 2. Methodology

### 2.1. Independent Component Analysis

Let *X* = [*x*_1_, *x*_2_,…, *x*_*m*_]^*T*^ be a matrix of size *m* × *n*, *m* ≤ *n*, consisting of observed mixture signals *x*_*i*_ of size 1 × *n*, *i* = 1,2,…, *m*. In the basic ICA model, the matrix *X* can be modeled as [17]

(1) $$\begin{array}{c} X=AS=\overset{m}{\underset{i=1}{\sum }}a_{i}s_{i}, \end{array}$$

where *a*_*i*_ is the *i*th column of the *m* × *m* unknown mixing matrix *A*; *s*_*i*_ is the *i*th row of the *m* × *n* source matrix *S*. The vectors *s*_*i*_ are latent source signals that cannot be directly observed from the observed mixture signals *x*_*i*_. The ICA model aims at finding an *m* × *m* demixing matrix *W* such that *Y* = [*y*_*i*_] = *WX*, where *y*_*i*_ is the *i*th row of the matrix *Y*, *i* = 1,2,…, *m*. The vectors *y*_*i*_ must be as statistically independent as possible and are called independent components (ICs). When demixing matrix *W* is the inverse of mixing matrix *A*, that is, *W* = *A* ^−1^, ICs (*y*_*i*_) can be used to estimate the latent source signals *s*_*i*_. Several existing algorithms can be used to perform ICA modeling.

The ICA modeling is formulated as an optimization problem by setting up the measure of the independence of ICs as an objective function and using some optimization techniques for solving the demixing matrix *W*. Several existing algorithms can be used to perform ICA modeling [17]. In general, the ICs are obtained by using the demixing matrix *W* to multiply the matrix *X*; that is, *Y* = *WX*. The demixing matrix can be determined using an unsupervised learning algorithm with the objective of maximizing the statistical independence of ICs. The ICs with non-Gaussian distributions imply the statistical independence, and the non-Gaussianity of the ICs can be measured by the negentropy [17]

(2) $$\begin{array}{c} J\left(y\right)=H\left(y_{\text{gauss}}\right)-H\left(y\right), \end{array}$$

where **y**_gauss_ is a Gaussian random vector having the same covariance matrix as **y**. *H* is the entropy of a random vector **y** with density *p*(**y**) defined as *H*(**y**) = −∫*p*(**y**)log⁡*p*(**y**)*d* **y**.

The negentropy is always nonnegative and is zero if and only if **y** has a Gaussian distribution. Since the problem in using negentropy is computationally very difficult, an approximation of negentropy is proposed as follows [17]:

(3) $$\begin{array}{c} J\left(y\right)\approx {\left[E\left\{G\left(y\right)\right\}-E\left\{G\left(v\right)\right\}\right]}^{2}, \end{array}$$

where *v* is a Gaussian variable of zero mean and unit variance, and *y* is a random variable with zero mean and unit variance. *G* is a nonquadratic function and is given by *G*(*y*) = exp⁡(−*y* ^2^/2) in this study. The FastICA algorithm proposed by Hyvärinen and Oja [17] is adopted in this paper to solve for the demixing matrix.

### 2.2. Support Vector Regression

Support vector regression (SVR) can be expressed as the following equation:

(4) $$\begin{array}{c} f\left(x\right)=\left(z\cdot \phi \left(x\right)\right)+b, \end{array}$$

where *z* is weight vector, *b* is bias, and *ϕ*(*x*) is a kernel function which use a nonlinear function to transform the nonlinear input to be linear mode in a high dimension feature space.

Traditional regression gets the coefficients through minimizing the square error which can be considered as empirical risk based on loss function. Vapnik [22] introduced so-called *ε-*insensitivity loss function to SVR. It can be expressed as follows:

(5) $$\begin{array}{c} L_{\varepsilon }\left(f\left(x\right)-q\right)=\{\begin{array}{cc} \left|f\left(x\right)-q\right|-\varepsilon & \text{if}\;\left|f\left(x\right)-q\right|\geq \varepsilon \\ 0 & \text{otherwise}, \end{array} \end{array}$$

where *q* is the target output; *ε* defined the region of *ε-*insensitivity; when the predicted value falls into the band area, the loss is zero. Contrarily, if the predicted value falls out the band area, the loss is equal to the difference between the predicted value and the margin.

Considering empirical risk and structure risk synchronously, the SVR model can be constructed to minimize the following programming:

(6) Min:    12zTz+C∑i(ξi+ξi∗)Subject  to qi−zTxi−b≤ε+ξi       zTxi+b−qi≤ε+ξi∗       ξi,ξi∗≥0,

where *i* = 1,2,…, *n* is the number of training data; (*ξ*_*i*_ + *ξ*_i_*) is the empirical risk; (1/2)*z* ^*T*^ *z* is the structure risk preventing overlearning and lack of applied universality; *C* is modifying coefficient representing the trade-off between empirical risk and structure risk. Equation (6) is a quadratic programming problem. After selecting proper modifying coefficient (*C*), width of band area (*ε*), and kernel function (*K*), the optimum of each parameter can be resolved though Lagrange function. The general form of the SVR-based regression function can be written as follows [22]:

(7) $$\begin{array}{c} f\left(x,z\right)=f\left(x,\alpha ,\alpha^{*}\right)=\overset{N}{\underset{i=1}{\sum }}\left(\alpha_{i}-\alpha_{i}^{*}\right)K\left(x,x_{i}\right)+b, \end{array}$$

where *α*_*j*_ and *α*_j_* are Lagrangian multipliers and satisfy the equality *α*_*j*_ *α*_j_* = 0; *K*(*x*_*i*_, *x*_*i*_′) is the kernel function. Any function that meets Mercer's condition can be used as the kernel function.

Although several choices for the kernel function are available, the most widely used kernel function is the radial basis function (RBF) defined as [36] *K*(*x*_*i*_, *x*_*j*_) = exp⁡(−||*x*_*i*_−*x*_*j*_||^2^/2*σ* ^2^), where *σ* denotes the width of the RBF. Thus, the RBF with parameter *σ* = 0.2 is applied in this study as kernel function.

SVR performance is mainly affected by the setting of parameters *C* and *ε* [36]. There are no general rules governing the choice of *C* and *ε*. The grid search proposed by Lin et al. [37] is a common and straightforward method using exponentially growing sequences of *C* and *ε* to identify good parameters (e.g., *C* = 2^−15^, 2^−13^, 2^−11^,…, 2^15^). The parameter set of *C* and *ε* which generate the minimum forecasting mean square error (MSE) is considered as the best parameter set. In this study, the grid search is used in each cluster to determine the best parameter set for training an optimal SVR forecasting model.

## 3. The Proposed Sales Forecasting Scheme

In this study, a hybrid sales forecasting scheme by combining ICA, K-means, and SVR is proposed. The proposed model contains three stages. In the first stage, the ICA model is applied to the input data to estimate independent components (ICs), demixing matrix, and mixing matrix from input data. The ICs can be used to represent hidden information/features of the input data. After obtaining ICs, the aim of this stage is to find the relationship between ICs and observed input data using mixing matrix. In the second stage, the K-means clustering algorithm groups the input data into several disjoined clusters using the mixing matrix. Each cluster contains similar objects. In the third stage, for each cluster, the SVR forecasting model is constructed and the final forecasting results can be obtained.

The detailed procedure of the proposed scheme is as follows.

(1) Consider *M* observed company sales data *x*_*i*_, *i* = 1,2,…, *M*, each of size 1 × *t*. And then combine *x*_*i*_ as an input data matrix *X*, *X* = [*x*_1_, *x*_2_,…, *x*_*M*_]^*T*^, of size *M* × *t*.
(2) Compute the demixing matrix *W* = [*w*_1_, *w*_2_,…, *w*_*M*_]^*T*^ of size *M* × *M* and independent component matrix *Y* = [*y*_1_, *y*_2_,…, *y*_*M*_]^*T*^ of size *M* × *t* using ICA method to the matrix *X*; that is, *Y* = *WX*. The row vectors *y*_*i*_ are ICs.
(3) As mentioned in Section 2, the ICs (*y*_*i*_) can be used to estimate the latent source signals (or hidden features) *s*_*i*_ which are the rows of the unknown source matrix *S* of size *M* × *t*. Thus, according to (1), the observed data matrix *X* can be obtained by using mixing matrix *A*, *A* = *W* ^−1^, to multiply the independent component matrix *Y*; that is, *X* = *AS* = *W* ^−1^ *Y* = *AY*.
(4) For each row vector *x*_*i*_, it can be obtained by using the corresponding row vector of matrix *A*, *a*_*i*_, to multiply the matrix *Y*; that is, *x*_*i*_ = *a*_*i*_ *Y*. Thus, the relationship between each observed company sales data *x*_*i*_ and the ICs can represented as
  (8) $$\begin{array}{c} x_{i}=a_{i1}y_{1}+a_{i2}y_{2}+\cdots +a_{iM}y_{M},\;i=1,2,\ldots ,M. \end{array}$$
(5) From (5), it can be found that each vector *a*_*i*_ can be used to represent the effects of different ICs on information (or features) contained in the corresponding sales data *x*_*i*_. Thus, the mixing matrix *A*, *A* = [*a*_1_, *a*_2_,…, *a*_*M*_]^*T*^, can be used for clustering since any two similar sales data *x*_*i*_ and *x*_*j*_, *i* ≠ *j*, will have similar patterns in the vectors *a*_*i*_.
(6) Use the K-means clustering algorithm to the mixing matrix *A* for clustering the observed sales data (i.e., the companies) into several disjointed clusters.
(7) Multiple SVR models that best fit each cluster are constructed by finding the optimal learning parameters of SVRs. Every constructed SVR model is the most adequate one for a particular cluster.
(8) For each company, find the cluster which it belongs to and use the SVR model corresponding to the cluster to generate final forecasting results.

## 4. Experimental Results

In order to evaluate the performance of the proposed hybrid sales forecasting scheme using ICA, K-means, and SVR algorithms (called ICA-k-SVR model), the monthly sales data of an IT product agent in Taiwan are used in this study. The monthly sales data of 30 companies from July 2003 to February 2010 are collected. For each company, there are a total of 80 data points in the dataset. The dataset is divided into training and testing datasets. The first 56 data points (70% of the total sample points) are used as the training samples while the remaining 24 data points (30% of the total sample points) are employed as the holdout and used as the testing sample for measuring out-of-sample forecasting ability. In the dataset, the agent classifies the 30 companies into three groups: Manufacturing; Service; and Finance sectors, according the business type of each company. Note that the assigned group of each company is viewed as its original group.  Table 1 shows the basic statistics of the data of each group. From Table 1, it can be seen that the sales data in the Finance sector is the most dynamic since it has the highest average, standard deviation, and maximum sales amount, as well as the lowest minimum sales amount.

The prediction results of the proposed sales forecasting scheme are compared to that of three other forecasting schemes, that is, single-SVR, K-means-SVR, and ICA-GHSOM-SVR schemes. In the single-SVR scheme, the SVR models are directly applied to each original group for building sales forecasting model. That is, in this study, three SVR sales forecasting models are individually constructed for the three groups. In the K-means-SVR scheme, the K-means algorithm is first used to cluster the 30 firms according to the patterns of the original sales data and then the SVR is utilized to build sales forecasting models for each group. In constructing the ICA-GHSOM-SVR model, the parameter setting and modeling process is the same as that described in Lu and Wang [11]. For more detailed information about the ICA-GHSOM-SVR model, please refer to Lu and Wang [11].

In this study, all of the four forecasting schemes are used for one-step-ahead forecasting of monthly sales data. In building the SVR forecasting model, the LIBSVM package proposed by Chang and Lin [38] is adapted in this study. The original datasets are first scaled into the range of [−1.0, 1.0] when using the LIBSVM package. Three forecasting variables including the previous one month's sales amount, previous two months' sales amount, and previous three months' sales amount are used for SVR modeling.

The prediction performance is evaluated using the following performance measures, namely, the root mean square error (RMSE), mean absolute difference (MAD), and mean absolute percentage error (MAPE). The smaller are the values of RMSE, MAD, and MAPE, the closer are the predicted results to that of the actual value. The definitions of these criteria are as follows:

(9) $$\begin{array}{c} \text{RMSE}=\sqrt{\frac{\sum_{i=1}^{n}{\left(T_{i}-P_{i}\right)}^{2}}{n}},\\ \text{MAD}=\frac{\sum_{i=1}^{n}{\left|T_{i}-P_{i}\right|}^{2}}{n},\\ \text{MAPE}=\frac{\sum_{i=1}^{n}\left|\left(T_{i}-P_{i}\right)/T_{i}\right|}{n}, \end{array}$$

where *T*_*i*_ and *P*_*i*_ represent the actual and predicted value at week *i*, respectively; *n* is the total number of data points.

This study used three clustering-based models for sales forecasting. The K-means algorithm in the K-means-SVR model directly uses the original values of the data points as input variables. In the ICA-GHSOM-SVR model and proposed ICA-k-SVR model, the GHSOM and K-means algorithms employ the elements of mixing matrix as inputs, respectively. Note that the clusters of companies generated by the K-means or GHSOM algorithms of the three clustering-based models are the temporary clusters which are used to build more effective forecasting models. For comparing the forecast results of the single-SVR model, each company clustered by the K-means algorithms of the three clustering-based models is reallocated to its original cluster. After reassigning all classified companies to their original group, the prediction errors of the three clustering-based models are then recalculated based on the original grouping results. For example, as seen in Table 2, the K-means algorithm clusters both company #1 and company #13 into Cluster 2. Based on the relationship depicted in Table 2, company #1 and company #13 can be reassigned to the Manufacturing sector and the Service sector, respectively.

Table 3 shows the forecasting results of each group defined by the IT agent, applying the individual SVR model to each group. Note that the best parameter sets of the SVR models for the Manufacturing, Service, and Finance sectors are (*C* = 2^3^, *ε* = 2^−3^), (*C* = 2^−1^, *ε* = 2^−7^), and (*C* = 2^1^, *ε* = 2^−1^), respectively. Table 3 shows that the single SVR model performs well in the Service sector but generates a higher forecasting error in the Finance sector.

Tables 4, 5, and 6 show the forecasting results of each group defined by the IT product agent by applying K-means-SVR model, ICA-GHSOM-SVR model, and the proposed ICA-k-SVR sales forecasting scheme, respectively. The best parameter sets of the K-means-SVR, ICA-GHSOM-SVR, and ICA-k-SVR models for the three sectors are summarized in Table 7.

In order to compare the performance of the four methods, Table 8 summarizes the forecasting results of Manufacturing, Service, and Finance using the four models. It can be observed from Table 8 that the proposed sales forecasting scheme produces the best forecasting results and outperforms the other three methods in all groups. Thus, it indicates that the proposed sales forecasting scheme provides a better forecasting result than the three comparison models in terms of prediction error.

Moreover, it also can be observed from Table 8 that K-means algorithm is successfully applied for grouping companies using sales data since the forecasting performance of K-means-SVR and the proposed forecasting schemes are better than the single-SVR model. As the ICA-GHSOM-SVR and the proposed model can generate better forecasting results, the ICA model is an effective feature extraction model and can improve the performance of the forecasting model in this study.

## 5. Conclusions

This paper proposed a hybrid sales forecasting scheme using ICA, K-means, and SVR for IT product agent. The proposed scheme, first, uses the ICA to extract hidden/underlying information (i.e., features) from the observed sales data. The extracted features are then applied to K-means algorithm for clustering the sales data into several disjoined clusters. Finally, for each cluster, the SVR forecasting model is constructed and final forecasting results are obtained. The monthly sales data collected from an IT product agent are used in this study for evaluating the performance of the proposed method. Experimental results showed that the proposed sales forecasting scheme produces the best forecasting results and outperforms the three comparison methods. According to the experiments, it can be concluded that the K-means algorithm is a promising tool for grouping companies using sales data and ICA model is an effective feature extraction model. The proposed hybrid sales forecasting scheme is an effective alternative for sales forecasting.

## Figures and Tables

**Table 1:** Basic statistics of the data in each sector.

Group name	Number of companies	Mean	Standard deviation	Max	Min
Manufacturing	12	413.23	321.01	10201.32	2.32
Service	10	291.01	223.45	7723.45	1.21
Finance	8	762.25	1191.32	18646.55	0.41

*Unit: thousand NT dollars.

**Table 2:** Comparison of the K-means clustering results and the original grouping results.

Company	Original group	K-means group
#1	Manufacturing	**2**
#2	Manufacturing	**1**
#3	Manufacturing	**3**
#4	Manufacturing	**1**
#5	Manufacturing	**1**
#6	Manufacturing	**2**
#7	Manufacturing	**2**
#8	Manufacturing	**1**
#9	Manufacturing	**3**
#10	Manufacturing	**3**
#11	Manufacturing	**2**
#12	Manufacturing	**1**
#13	Service	**2**
#14	Service	**2**
#15	Service	**3**
#16	Service	**1**
#17	Service	**1**
#18	Service	**1**
#19	Service	**2**
#20	Service	**2**
#21	Service	**2**
#22	Service	**3**
#23	Finance	**1**
#24	Finance	**1**
#25	Finance	**1**
#26	Finance	**2**
#27	Finance	**3**
#28	Finance	**2**
#29	Finance	**3**
#30	Finance	**1**

**Table 3:** The forecasting results of each group by using the single-SVR model.

Group name	Number of companies	RMSE	MAD	MAPE
Manufacturing	12	426.28	203.84	3.27%
Service	10	293.76	96.07	3.11%
Finance	8	1488.69	499.99	4.72%

**Table 4:** The forecasting results of each group by using the K-means-SVR model.

Group name	Number of companies	RMSE	MAD	MAPE
Manufacturing	12	350.28	132.21	1.89%
Service	10	222.95	62.12	2.23%
Finance	8	851.85	378.81	3.71%

**Table 5:** The forecasting results of each group by using the ICA-GHSOM-SVR model.

Group name	Number of companies	RMSE	MAD	MAPE
Manufacturing	12	278.31	105.90	1.29%
Service	10	109.86	46.87	1.76%
Finance	8	593.76	268.32	3.57%

**Table 6:** The forecasting results of each group by using the proposed sales forecasting model.

Group name	Number of companies	RMSE	MAD	MAPE
Manufacturing	12	252.12	92.90	0.96%
Service	10	84.22	27.12	1.31%
Finance	8	445.19	201.91	3.34%

**Table 7:** Summary of best parameter sets of the K-means-SVR, ICA-GHSOM-SVR, and ICA-k-SVR for the three sectors.

Group name	Models	Best parameter sets
K-means-SVR	Manufacturing	(*C* = 2^−1^, ε = 2^−1^)
	Service	(*C* = 2^−5^, ε = 2^−3^)
	Finance	(*C* = 2^5^, ε = 2^−5^)

ICA-GHSOM-SVR	Manufacturing	(*C* = 2^3^, ε = 2^1^)
	Service	(*C* = 2^3^, ε = 2^3^)
	Finance	(*C* = 2^−1^, ε = 2^−1^)

ICA-k-SVR	Manufacturing	(*C* = 2^−7^, ε = 2^−3^)
	Service	(*C* = 2^−9^, ε = 2^−3^)
	Finance	(*C* = 2^−5^, ε = 2^−5^)

**Table 8:** Summary of forecast results by the four models.

Group name	Models	MAD	RMSE	MAPE
Manufacturing	single-SVR	426.28	203.84	3.27%
	K-means-SVR	350.28	132.21	1.89%
	ICA-GHSOM-SVR	278.31	105.90	1.29%
	**ICA-k-SVR**	**252.12 **	**92.90 **	**0.96%**

Service	single-SVR	293.76	96.07	3.11%
	K-means-SVR	222.95	62.12	2.23%
	ICA-GHSOM-SVR	109.86	46.87	1.76%
	**ICA-k-SVR**	**84.22 **	**27.12 **	**1.31%**

Finance	single-SVR	1488.69	499.99	4.72%
	K-means-SVR	851.85	378.81	3.71%
	ICA-GHSOM-SVR	593.76	268.32	3.57%
	**ICA-k-SVR**	**445.19 **	**201.91 **	**3.34%**
